# Navigating tensions in climate change-related planned relocation

**DOI:** 10.1007/s13280-024-02035-2

**Published:** 2024-06-07

**Authors:** Giovanna Gini, Annah Piggott-McKellar, Hanne Wiegel, Friedrich Nikolaus Neu, Ann-Christine Link, Claudia Fry, Tammy Tabe, Olumuyiwa Adegun, Cheikh Tidiane Wade, Erica Rose Bower, Sarah Koeltzow, Rachel Harrington-Abrams, Carolien Jacobs, Kees van der Geest, Narjes Zivdar, Ryan Alaniz, Carolyne Cherop, David Durand-Delacre, Melanie Pill, Himanshu Shekhar, Olivia Yates, Md Abdul Awal Khan, Frank Kwesi Nansam-Aggrey, Lauren Grant, Danang Aditya Nizar, Kwame Nitri Owusu-Daaku, Alberto Preato, Oana Stefancu, Merewalesi Yee

**Affiliations:** 1 School of Geography and the Environment, University of Oxford, Oxford, UK; 2 South American Network for Environmental Migrations (RESAMA), São Paulo, Brazil; 3 School of Architecture and Built Environment, Queensland University of Technology, Brisbane, Australia; 4 Geography, Earth and Atmospheric Sciences, Melbourne University, Parkville, Australia; 5 Environmental Policy Group and the Sociology of Development and Change Group, Wageningen University, Wageningen, The Netherlands; 6 Chair Group of Geography of Global Change, Institute of Environmental Social Sciences and Geography, University of Freiburg, Freiburg, Germany; 7 Department of Social Inquiry, La Trobe University, Melbourne, Australia; 8 United Nations University Institute for Environment and Human Security, Bonn, Germany; 9 Department of Geography, Philipps-Universität Marburg, Marburg, Germany; 10 Department of Geography, University of Exeter, Exeter, UK; 11 Research Program, East-West Centre, Honolulu, HI USA; 12 Department of Architecture, Federal University of Technology, Akure, Nigeria; 13 School of Architecture and Planning, University of the Witwatersrand, Johannesburg, South Africa; 14 Department of Geography, Assane Seck University, Ziguinchor, Senegal; 15 Doerr School of Sustainability, Stanford University, Palo Alto, CA USA; 16 Platform On Disaster Displacement Secretariat, Geneva, Switzerland; 17 Department of Geography, King’s College London, London, UK; 18 Van Vollenhoven Institute for Law, Governance and Society, Leiden University, Leiden, The Netherlands; 19 United Nations High Commissioner for Refugees, UNHCR, Tehran, Iran; 20 Social Sciences Department, Cal Poly State University, San Luis Obispo, CA USA; 21 Climate Desk, Parliament of Kenya, Nairobi, Kenya; 22 Indo-Pacific Development Centre, Lowy Institute, Sydney, Australia; 23 School of Psychology, The University of Auckland, Auckland, New Zealand; 24 Department of Law, Independent University, Bangladesh, Dhaka, Bangladesh; 25 Climate Change Department, National Disaster Management Organization, Accra, Ghana; 26 International School On Climate Migration and Earth Refuge, London, UK; 27 Raoul Wallenberg Institute of Human Rights and Humanitarian Law, Regional Asia Pacific Office, Jakarta, Indonesia; 28 Department of Earth and Environmental Sciences, University of West Florida, Pensacola, FL USA; 29 United Nations Human Settlements Programme, UN-Habitat, Nairobi, Kenya; 30 School of the Environment, University of Queensland, Brisbane, Australia

**Keywords:** Climate adaptation, Climate justice, Climate risk, Disaster risk reduction, Loss and damage, Managed retreat

## Abstract

The planned relocation of communities away from areas of climate-related risk has emerged as a critical strategy to adapt to the impacts of climate change. Empirical examples from around the world show, however, that such relocations often lead to poor outcomes for affected communities. To address this challenge, and contribute to developing guidelines for just and sustainable relocation processes, this paper calls attention to three fundamental tensions in planned relocation processes: (1) conceptualizations of risk and habitability; (2) community consultation and ownership; and (3) siloed policy frameworks and funding mechanisms. Drawing on the collective experience of 29 researchers, policymakers and practitioners from around the world working on planned relocations in the context of a changing climate, we provide strategies for collectively and collaboratively acknowledging and navigating these tensions among actors at all levels, to foster more equitable and sustainable relocation processes and outcomes.

## Introduction

2023 was the hottest year in recorded history (WMO 2024). The 1.5 °C degree threshold identified in the Paris Agreement as the desired ‘maximum’ of planetary warming is projected to be surpassed anytime from this year, 2024, to the early 2030s (Jones 2023; Milman 2024). While climate impacts are already being experienced by millions of people globally, crossing this threshold will lead to: widespread heatwaves in many parts of the world; increased precipitation in high-latitude and mountainous regions; severe droughts affecting water availability; and sea-level rise in the coastal zones. These climatic extremes exacerbate vulnerabilities particularly for the poorest and most vulnerable populations and are calling into question the continued habitability of some places.

In this context, planned relocation of populations away from high-risk areas will very likely increase. Since the 1970s, over 400 planned relocations related to natural hazards, disasters and climate change have been identified across 78 countries (Bower et al. 2022). Planned relocation in the context of climate change refers to the coordinated, permanent movement of people from places that are, or soon will be, affected by acute climate impacts such as coastal and riverine flooding, melting permafrost, and by associated land loss. Planned relocation usually occurs at the community level, with the support of external actors under State authority, and within national borders (UNHCR et al. 2015; Ferris and Weerasinghe 2020).

The urgency in addressing planned relocation was underscored during the 28th Conference of the Parties (COP28) of the United Nations Framework Convention on Climate Change (UNFCCC), where human mobility driven by climate change, including planned relocation, consistently featured in side events and negotiations. This heightened recognition is now formally embedded in the decision text of the first global stocktake where, notably, planned relocation is specifically addressed. Despite the increase in planned relocations and global recognition of its importance, only Fiji (2018) and Solomon Islands (2022) have developed national climate change-related relocation guidelines. While global guidelines and principles exist (UNHCR et al. 2015; Georgetown University 2017), there is little consensus about where, when, and how planned relocation should occur in practice. This complicates the implementation of relocation processes, often to the detriment of the relocating population, and emphasizes the scale and urgency of the challenge ahead as the habitability thresholds of more places around the world are crossed.

In light of the need to address this issue, we as 29 researchers, practitioners, and policymakers from the 2023 Climate Academy on Planned Relocation^1^ argue that planned relocation processes create fundamental tensions understood as ideas or processes with conflicting demands or implications, between the involved actors. While these are inherent in relocation processes, they are often not sufficiently addressed in policy and practice, increasing the probability of undesired outcomes. Our argument is not that tensions are challenges that need to be overcome, but rather that through acknowledging and embracing tensions, they present a critical point of departure, and an opportunity to collaboratively forge more just and sustainable ways of doing planned relocation.

In this paper, we identify and unpack three tensions inherent in planned relocation: (1) conceptualizations of risk and habitability, (2) community consultation and ownership, and (3) siloed policy frameworks and funding mechanisms. We call for embracing these tensions as critical points of departure for collaboratively forging more just and sustainable relocation processes and provide strategies for navigating them by drawing on examples from around the world.

### Tension 1: Conceptualizations of risk and habitability

Decisions regarding whether communities should relocate typically center on defining the habitability thresholds of places. Despite recent advancements in the conceptualization of risks, these tend to be limited to biophysical risk assessments and often view uninhabitability as a predefined outcome. This can stand in stark contrast with the affected population’s knowledge of their environment, perceptions of risks and risk tolerance (Farbotko et al. 2023). The latter is shaped by worldviews that are reflected in people’s identities concerning their belonging, relations to others and their environment—and their resulting preferences on if and when to relocate.

If assessments of habitability do not proactively include how communities perceive and address risks in their daily lives and do not consider diverse perspectives, knowledge and ways of living of affected populations, they may result in problematic planned relocation decisions and processes. This may then exacerbate marginalization, feelings of discrimination and inequality, as well as the erosion of cultural and social capital, leading to further unintended negative consequences (Farbotko et al. 2020).

The case of Villa Santa Lucía, Chile, illustrates this tension. The local community rejected the government’s relocation plans after a mudslide in 2017, despite projections that mudslides are likely to occur more frequently under intensifying climate change. Based on specific understandings of nature and human–nature relations, the local population did not consider mudslides a risk that warranted abandoning their village (Wiegel et al. 2021).

Navigating this tension requires high levels of sensitivity when integrating differing worldviews and understandings of risk in relocation assessments. It requires the ability, willingness and flexibility of decision-makers to listen to and value affected populations' knowledge, opinions and preferences in relocation planning and translate these voices into tangible policy outcomes. An example of how this is being done is the Comprehensive Risk and Vulnerability Assessment Matrix by the Fijian Government that incorporates not only biophysical and climate data to assess risks but also a community-level social, economic and cultural assessment (Fiji Government 2023).

### Tension 2: Community consultation and ownership

Planned relocations typically involve governments as enabling actors, who should, according to existing guidelines, consult with the affected community (IPCC 2018; Ferris and Weerasinghe 2020; Bower et al. 2022; Milman 2024). In practice, however, consultation is often inadequate in terms of format, timing, and participation or is missing altogether. When a collaborative process is absent, perfunctory or rigidly designed to achieve predefined outcomes, it limits community ownership of the relocation process. This can both amplify feelings of loss and disruption and have negative repercussions for the continuity of livelihoods, identities, and well-being. The 1955–1971 Gilbertese resettlement to the Solomon Islands shows how a lack of community agency in the decision to move resulted in poor inter-generational outcomes (Tabe 2019). This highlights tensions around the roles and responsibilities of various actors, particularly regarding how and to what extent affected communities own the process and have de-facto decision-making power throughout different stages of the relocation.

Inadequate collaboration between the affected population and supporting actors may lead some communities to relocate independently or to not relocate at all. For example, faced with severe erosion, the Enseada da Baleia community on Cardoso Island, Brazil, refused the government's relocation plan. Instead, the community organized their own relocation, relying on traditional knowledge to protect their way of life and cultural traditions (Gini et al. 2020). While this exemplifies a community-driven response, it also underscores the financial, physical, and psychological burdens the Enseada community faced due to the lack of external funding and support.

Establishing collaborative, clear and long-term relationships among national governments, local authorities, affected communities, and non-state actors (including international organizations, religious groups, cultural organizations, and other community entities) is crucial to navigate this tension. Building these collaborative relationships takes time and should start long before any decision for planned relocation is made and continue throughout the planning and implementation process. Examples of positive collaborative processes within planned relocations are emerging and have seemingly achieved better outcomes for the community. For instance, the relocation of Cogea village in Fiji has embedded collaborative processes not only in the decision to move, but also with community input into the house design, including psychosocial support and building relationships with local organizations.

### Tension 3: Siloed policy frameworks and funding mechanisms

Funding and policy frameworks tend to approach planned relocation as either climate change adaptation, disaster risk reduction, or loss and damage, each involving different sets of institutions, implementation procedures, and operational responsibilities. Examples are the Global Compact for Safe, Orderly, and Regular Migration, the Sendai Framework for Disaster Risk Reduction, and the Warsaw International Mechanism for Loss and Damage associated with Climate Change Impacts.

The siloed nature of policy and funding domains can create several challenges when planning and implementing relocation processes. For example, communities that wish to relocate may not be prioritized until the situation becomes sufficiently critical to fall under a specific policy framework (i.e., loss and damage). This delay can lead to substantial yet avoidable losses, increase people’s vulnerabilities, or lead to populations undertaking autonomous relocation processes without adequate resources and support.

Further, funding is often short-term, and project-based. This cannot support the long-term and multi-sectoral planning needed to deal with the broad spectrum of challenges communities face before, during, and in particular after relocating. These include preserving or rebuilding cultural identities, rebuilding sustainable livelihoods, and enhancing long-term resilience to future shocks (Alaniz 2017). For example, after Cyclone Idai, people across Mozambique were displaced and provided shelter in tents by humanitarian actors, with plans to permanently relocate (Jacobs and Almeida 2021). However, more than four years later, relocation is still incomplete, with development-oriented actors now supporting the construction of durable houses.

To navigate this tension, funding and policy frameworks should address the multi-dimensional and long-term challenges associated with planned relocation by fostering collaboration and coordination between international, national, and local funding organizations and across policy domains. Instead of viewing planned relocation purely through one single siloed framework, gaps between them need to be bridged, and a multi-dimensional approach adopted. This needs to consider intersectional and often long-standing structural issues (e.g., poverty, uneven resource access) faced by the affected populations, as well as the long-term nature of relocation processes.

## Embracing tensions to forge sustainable and just solutions

Tensions are an inherent aspect of planned relocation. In this paper, we have focused on disentangling three tensions that, based on our collective experiences, we deem most critical. First, unpacking the ways risk and habitability are conceptualized presents an opportunity to engage in dialogue that integrates divergent worldviews and bridges different knowledge systems. Second, in asking who is responsible for which elements of planned relocation processes, there is potential to reframe flawed consultation processes and foster meaningful collaborative and long-term relationships between affected communities, governments, and non-governmental actors. Third, critically assessing the limitations of siloed policy and funding frameworks to support planned relocations can help foster solutions that support affected populations to deal with the many challenges they face and create long-term solutions.

As we confront the reality of a 1.5 °C warmer world, more communities urgently need to relocate away from high-risk climate areas. Practitioners, policymakers, and researchers must remain vigilant about the complexities and contradictions inherent in planned relocations. They must navigate these tensions effectively, acknowledging that these cannot be simply wished away.

## Footnotes

1 The Climate Academy 2023 was organized by the United Nations University Institute for Environment and Human Security and funded by Munich Re Foundation. The academy included online presentations and discussions from 11–15 September 2023, and an in-person workshop from 25–29 September 2023 in Saly, Senegal. More information on the Climate Academy 2023 can be found at https://www.munichre-foundation.org/en/climate-adaptation/the-climate-academy/CA2023-Downloads.html..
