# Correction to: Navigating tensions in climate change-related planned relocation

**DOI:** 10.1007/s13280-024-02059-8

**Published:** 2024-07-31

**Authors:** Giovanna Gini, Annah Piggott-McKellar, Hanne Wiegel, Friedrich Nikolaus Neu, Ann-Christine Link, Claudia Fry, Tammy Tabe, Olumuyiwa Adegun, Cheikh Tidiane Wade, Erica Rose Bower, Sarah Koeltzow, Rachel Harrington-Abrams, Carolien Jacobs, Kees van der Geest, Narjes Zivdar, Ryan Alaniz, Carolyne Cherop, David Durand-Delacre, Melanie Pill, Himanshu Shekhar, Olivia Yates, Md Abdul Awal Khan, Frank Kwesi Nansam-Aggrey, Lauren Grant, Danang Aditya Nizar, Kwame Nitri Owusu-Daaku, Alberto Preato, Oana Stefancu, Merewalesi Yee

**Affiliations:** 1 School of Geography and the Environment, University of Oxford, Oxford, UK; 2 South American Network for Environmental Migrations (RESAMA), São Paulo, Brazil; 3 School of Architecture and Built Environment, Queensland University of Technology, Brisbane, Australia; 4 Geography, Earth and Atmospheric Sciences, Melbourne University, Parkville, Australia; 5 Environmental Policy Group and the Sociology of Development and Change Group, Wageningen University, Wageningen, The Netherlands; 6 Chair Group of Geography of Global Change, Institute of Environmental Social Sciences and Geography, University of Freiburg, Freiburg, Germany; 7 Department of Social Inquiry, La Trobe University, Melbourne, Australia; 8 United Nations University Institute for Environment and Human Security, Bonn, Germany; 9 Department of Geography, Philipps-Universität Marburg, Marburg, Germany; 10 Department of Geography, University of Exeter, Exeter, UK; 11 Research Program, East-West Centre, Honolulu, HI USA; 12 Department of Architecture, Federal University of Technology, Akure, Nigeria; 13 School of Architecture and Planning, University of the Witwatersrand, Johannesburg, South Africa; 14 Department of Geography, Assane Seck University, Ziguinchor, Senegal; 15 Doerr School of Sustainability, Stanford University, Palo Alto, CA USA; 16 Platform On Disaster Displacement Secretariat, Geneva, Switzerland; 17 Department of Geography, King’s College London, London, UK; 18 Van Vollenhoven Institute for Law, Governance and Society, Leiden University, Leiden, The Netherlands; 19 United Nations High Commissioner for Refugees, UNHCR, Tehran, Iran; 20 Social Sciences Department, Cal Poly State University, San Luis Obispo, CA USA; 21 Climate Desk, Parliament of Kenya, Nairobi, Kenya; 22 Indo-Pacific Development Centre, Lowy Institute, Sydney, Australia; 23 School of Psychology, The University of Auckland, Auckland, New Zealand; 24 Department of Law, Independent University, Bangladesh, Dhaka, Bangladesh; 25 Climate Change Department, National Disaster Management Organization, Accra, Ghana; 26 International School On Climate Migration and Earth Refuge, London, UK; 27 Raoul Wallenberg Institute of Human Rights and Humanitarian Law, Regional Asia Pacific Office, Jakarta, Indonesia; 28 Department of Earth and Environmental Sciences, University of West Florida, Pensacola, FL USA; 29 United Nations Human Settlements Programme, UN-Habitat, Nairobi, Kenya; 30 School of the Environment, University of Queensland, Brisbane, Australia

**Correction to: Ambio** 10.1007/s13280-024-02035-2

In the original published article, the author names “Friedrich Nikolaus Neu, Cheikh Tidiane Wade and Alberto Preato” were incorrectly written as “Frederich Neu, Cheikh Wade and Alberto Praeto”.

Also the ORCIDs for the below authors were updated in this correction.

Giovanna Gini

Hanne Wiegel

Friedrich Nikolaus Neu

Ann-Christine Link

Claudia Fry

Tammy Tabe

Olumuyiwa Adegun

Cheikh Tidiane Wade

Erica Rose Bower

Sarah Koeltzow

Rachel Harrington-Abrams

Carolien Jacobs

Kees van der Geest

Narjes Zivdar

Ryan Alaniz

David Durand-Delacre

Melanie Pill

Himanshu Shekhar

Olivia Yates

Md Abdul Awal Khan

Frank Kwesi Nansam-Aggrey

Kwame Nitri Owusu-Daaku

Merewalesi Yee

The original article has been corrected.

